# Commentary: Baseline adiponectin concentration and clinical outcomes among patients with diabetes and recent acute coronary syndrome in the EXAMINE trial

**DOI:** 10.3389/fendo.2018.00138

**Published:** 2018-04-03

**Authors:** Jan Brož, Marek Brabec, Milan Kvapil, Jan Polák

**Affiliations:** ^1^Department of Internal Medicine, Second Faculty of Medicine, Charles University, Prague, Czechia; ^2^Institute of Computer Science, The Czech Academy of Sciences, Prague, Czechia; ^3^Czech Institute of Informatics, Robotics, and Cybernetics, Czech Technical University in Prague, Prague, Czechia; ^4^Department for the Study of Obesity and Diabetes, Third Faculty of Medicine, Charles University, Prague, Czechia

**Keywords:** adiponectin, type 2 diabetes mellitus, cardiovascular risk, acute coronary syndrome, all-cause mortality rate

Bergmark et al. published a study dealing with the relationship of cardiovascular (CV) risks and adiponectin levels (1). The paper analyses the plasma levels of adiponectin in acute coronary syndrome type 2 diabetes patients (ACS) enrolled in the study EXAMINE (alogliptin vs. placebo, adiponectin sampled at study baseline) where they were monitored for CV outcomes (but not adiponectin) for a period of 18 months (2). Bergmark et al. conclude that adiponectin concentration was independently positively associated with increased risk of death from CV causes, all-cause mortality, and hospitalization for heart failure. In the introduction and the discussion, the authors mention that the relation between the adiponectin level and CV outcomes has been investigated in several studies, nevertheless with heterogeneous results: in some of them, adiponectin is associated with an increased CV risk, while others conclude that it is a protective factor. This study results rank adiponectin among factors that may increase CV risk in ACS type 2 diabetes patients.

Despite the fact that the evaluation and discussion in the paper of Bergmark et al. is very complex, we would like to respectfully address the authors with at least two comments which we consider important.

The period after ACS is a time when patients usually experience many changes in their treatment regimen. A number of these changes, such as dietary adjustment, increased physical activity (3–5), new medicaments related to ACS (acetylsalicylic acid, some kinds of statins and beta-blockers), or those with regard to the metabolic control of diabetes (thiazolidinediones) influence adiponectin levels in different directions in weeks (6, 7).

If such changes were made in patients who were later enrolled in the EXAMINE study, which is highly probable, then the adiponectin plasma level at the study baseline was proportional to the time of their effect in these subjects. And since the time difference between ACS and the enrollment in the study varied substantially in individual patients (15–90 days), we believe that this fact could lead to a significant distortion of the real adiponectin levels observed in individual patients during the whole study period which were then subject to analysis.

We understand that a detailed analysis of the above factors would be complicated, even though the data on medication are certainly available in the study. Nevertheless, due to the importance of the study results and the fact that it may be assumed that patients were treated taking into account the above stated parameters following the current guidelines, consequently quite similarly, we would recommend making an adjustment of adiponectin levels, taking into account the elapsed time from the coronary event (e.g., employing linear model or better, a GAM) and test the outcomes against the thus obtained results (in a manner analogous to the standard ANCOVA analysis).

Our second comment concerns the possibility of a more detailed study dealing with the effect of initial adiponectin values rather than with the discretization into quartiles. A quantitative view based on the estimation of functional dependence between CV outcomes and adiponectin (e.g., employing Generalized Additive Model with splines that allow for modeling of a possible nonlinearity). If the shape of CV response to the increasing adiponectin concentration was realistic (which is not known from the quartiles comparison done so far), it would certainly add weight to the evidence provided by Bergmark et al. in their publication.

## Author Contributions

JB designed and wrote the commentary. MB, MK, and JP contributed to the design and revised the text critically for important intellectual content.

## Conflict of Interest Statement

The authors declare that the research was conducted in the absence of any commercial or financial relationships that could be construed as a potential conflict of interest.
